# Cage egg producers' perspectives on the adoption of cage-free systems in China, Japan, Indonesia, Malaysia, Philippines, and Thailand

**DOI:** 10.3389/fvets.2022.1038362

**Published:** 2022-12-05

**Authors:** Maria Catalina Tan de Luna, Qing Yang, Ali Agus, Shuichi Ito, Zulkifli Idrus, Rahayu H. S. Iman, Jutamart Jattuchai, Elissa Lane, Jayasimha Nuggehalli, Kate Hartcher, Michelle Sinclair

**Affiliations:** ^1^Department of Basic Veterinary Sciences, University of the Philippines, Los Baños, Philippines; ^2^Royal (Dick) School of Veterinary Studies, The University of Edinburgh, Edinburgh, United Kingdom; ^3^Global Food Partners, Singapore, Singapore; ^4^Faculty of Animal Science, Gadjah Mada University, Yogyakarta, Indonesia; ^5^School of Agriculture, Tokai University, Kumamoto, Japan; ^6^Institute of Tropical Agriculture and Food Security, Universiti Putra Malaysia, Seri Kembangan, Malaysia; ^7^Fakultas Peternakan, Bogor Agricultural University, Bogor, Indonesia; ^8^Faculty of Veterinary Science, Chulalongkorn University, Bangkok, Thailand; ^9^NALSAR University of Law, Hyderabad, India; ^10^School of Veterinary Science, University of Queensland, Brisbane, QLD, Australia; ^11^Animal Law and Policy Program, Harvard Law School, Cambridge, MA, United States

**Keywords:** animal welfare, chickens, hens, egg, production, Asia

## Abstract

Asia is responsible for ~60% of global egg production. As in most of the world, nearly all of the egg-laying hens are housed in cages. While there is growing demand for cage-free eggs in many regions of the world, challenges have been reported when transitioning to these systems, which may affect the willingness of producers to transition. The aim of this research was to investigate the views of Asian egg producers on the feasibility of cage-free systems and what they perceive to be the main challenges and proposed solutions in adopting cage-free systems. A total of 224 egg producers (165 cage egg producers) completed questionnaires containing a mix of free-form, Likert scale and demographic items. Data were analyzed using thematic qualitative analysis and descriptive quantitative statistics. Responses indicated that cages are primarily used for their efficiency and ease of management. The most common reasons to consider adopting cage-free systems included improved animal welfare, increased market access, and increased product quality. A majority of producers (65%) responded “yes” or “maybe” when asked if they consider cage-free systems to be feasible in their country. Perceived challenges in adopting cage-free systems included reduced profitability, higher costs, and biosecurity and disease. Potential solutions included the development of the cage-free industry and market development. Most producers (72%) said more support is needed to establish cage-free farms, mostly pertaining to technical advice, training and resources. The findings of this study provide an enhanced understanding of the egg industry in these countries and potential areas for producer support in transitioning to cage-free systems.

## Introduction

As of 2018, the continent of Asia was responsible for the production of 822 billion chicken eggs annually; 60% of total world production and was home to at least 3.1 billion egg-laying chickens (1). As is the case in most areas of the world, almost all of the hens are kept in cage production systems (1–3). Chicken and egg production arguably began in Asia, with the domestication of jungle fowl in natural open range farming environments (4). The industrialization of animal agriculture, coupled with the need to provide protein for growing populations, has facilitated the growth of the egg industry, unrivaled in the rest of the world.

Constituents and consumers around the world increasingly care about animal welfare and expect improved treatment and conditions for farm animals (5). Since the intensification of animal agriculture and the rise of affluence in Asia, widespread domestic poverty in countries such as China is rapidly becoming an epidemic of the past (6). Recent research has shown that “animal welfare” and “animal protection” are considered important in many countries in Asia amongst the general public (7–9), in agricultural science (10), and the livestock industry (11). One of the few studies on this topic that was conducted in the region, found that livestock industry leaders across Asia see a variety of benefits in improving animal welfare, such as; improved productivity, improved product quality, and market differentiation (12), and another study indicated that engaging industry stakeholders could be effective in improving industry practices and animal welfare (13).

There is growing demand for cage-free eggs from food businesses and consumers in Asia, and producers are looking to meet this demand by adopting cage-free systems (14). As such, cage-free egg production systems are currently emerging across the region (15). However, Asia and other regions of the world still primarily utilize cage-based systems of egg production; ~90% of eggs produced in China, 80% in India, and almost 100% of eggs produced in Malaysia are produced in cages (9). The risk of negative economic implications, such as an increase in the cost of production resulting in higher egg prices (16), and a perceived reduction in the hygiene of cage-free eggs (17), could serve to undermine the transition to cage-free systems. A recent study in China supports this presumption, where cage egg producers considered that a transition to cage-free systems would represent a financial loss (3). The exact nature of financial implications and challenges to the perspective of egg producers in China, and many other nations, is yet to be investigated and evaluated in any depth.

The primary goal for the present research was to investigate, from the producers' perspective, the perceived feasibility of cage-free systems as well as the main challenges egg producers face in adopting and maintaining cage-free egg farms, and some potential solutions across six key countries in the region: China, Indonesia, Japan, Malaysia, Philippines and Thailand. The key questions focused on: (1) the reasons to use conventional cage systems; (2) the perceived reasons to use cage-free systems; (3) whether cage-free systems are an option; (4) the perceived challenges in adopting cage-free systems; (5) potential solutions to the perceived challenges; (6) whether more support would be needed when adopting cage-free systems; (7) what support is needed; and (8) who should offer that support. The findings of this study are anticipated to provide an enhanced understanding of the industry in the focus countries and offer insight into potential areas for initiatives to support the egg industry in these countries in the transition from cage to cage-free systems of egg production.

## Methods

### Research ethics

This research was granted ethics approval through the University of Queensland Human Ethics Committee (#2020002225). Data collection was conducted between January and June 2021.

### Participants

Egg producers were eligible to participate in this study if they nominated their consent on the questionnaire, met the criteria in Table 1, and were deemed to have a working knowledge of their operation. Eligibility criteria was based on samples deemed representative of local industries in each country, rather than analogous criteria across all countries, as the nature of egg production industries vary by country. The countries selected for investigation in this study were selected for this inherent diversity in nature of production, diversification of culture and geographic distribution around Asia. Efforts were made to harmonize criteria where the scale of the industry allows, however the scale of the industries in each country did not allow for this. For example, farms tend to be no more than 50,000 hens in Indonesia, as compared to farms that commonly start at a size of 50,000 hens in China. As this area has scarcely been researched, and there does not exist a central repository for information in relation to cage-egg farms in any of these countries, the size of farm that was considered respectively “representative” was ascertained through consultation with local experts in each instance. In this nature, the perceptions reported in this study are representative of local industries, and findings are commonly delineated by country. Where similarities are found across countries and represented as aggregates, it could be considered that those perceptions may represent egg producers in Asia more broadly. Producers were approached by in-country academic collaborators (co-authors) based on their eligibility, which was ascertained by familiarly with their enterprise (including online research), and through network referrals. Eligibility and consent was re-established at the onset of participation in the study.

**Table 1:** Participant eligibility criteria.

Cage producers	Farm size	Representative of the size of cage farms in each country*
	Farming system	Conventional cages
	Role	Engaged in a role that has sufficient power within the organization to make or contribute to decisions on transitioning to cage-free, and knowledge of the operation.
	Length of service	Must have been working within the industry for a minimum of 1 year.
Cage-free producers	Farm size	Minimum 10,000 hens
	Farming system	Any cage free system. If farms have both cage and cage-free operations, they will be interviewed as cage-free.
	Role	Engaged in a role that requires a technical awareness of on-farm operations, including the challenges and benefits of operating within the cage-free egg production system.
	Length of service	Must have been working within the industry for a minimum of 1 year.
^*^Industry representative sample by country, as below:		
**Country**		**Farm size (number of hens)**
China		50,000+
Indonesia		10,000–50,000
Japan		500,000–1 million
Malaysia		50,000–500,000
Philippines		15,000–1 million
Thailand		50,000–500,000

### Research tool

Quantitative surveys are not always sufficient in investigating human attitudes and concerns (18) or in providing a “deeper” understanding of social phenomena (19). For this reason, a mixed methodology approach was adopted, with a primary emphasis on qualitative items.

Study information and an invitation to participate were prepared in local languages and sent ***via*** email to egg producers in China, Indonesia, Japan, Malaysia, Philippines, and Thailand. If the producers agreed to participate, they were provided with a link to an online questionnaire in their local language (Chinese, Bahasa Indonesia, Japanese, English, or Thai) to complete at a time that suited them. Responses were anonymous and were translated from the local language to English by translators proficient in each language for data analyses. Anonymity also served to protect data collected within this study, and raw and collated data were kept digitally and password protected. Separate questionnaires were developed for cage and cage-free producers, and the relevant questionnaire link was distributed depending on the production system used. A total of 20 questions, plus demographic and farm details, were asked across the questionnaires. Definitions of cage and cage-free production, as it pertains to this study, were offered to both cage and cage-free producers as follows;

Cage systems—The use of wire cages to house laying hens inside sheds.

Cage-free systems—Housing that does not use cages and in which the hens can move freely throughout a shed. Cage-free systems include free-range or indoor systems and can have one or more levels (aviaries).

The specific questions relevant to this paper asked the following:

- Most egg farmers in your country and around the world use cages. What are the reasons for using cages compared to cage-free systems? (Open-ended)
- Some egg farmers are changing to cage-free systems. What do you think are the reasons to use cage-free compared to cage systems? (Open-ended)
- Do you think cage-free systems are an option in your country? (Yes/No option)
- What do you think are the biggest challenges and problems that prevent cage farmers from using cage-free systems? (Open-ended)
- If an egg farmer in your country decided to use a cage-free system what would be some of the solutions to the challenges (outlined in Q4 above)?
- If an egg farmer decided to use a cage-free system, would they need more support in the establishment or maintenance of the farm than is currently available? (Yes/No option)
- What support would they need? (Open-ended)
- Who should offer that support? (Open-ended).

### Data analysis

The data were compiled, coded and cleansed, whereby responses that were abandoned by participants were removed and data columns were aligned for all countries to correspond with each question. All responses that were translatable were included in the analysis. Binary and numerical data were summarized and qualitative data was subjected to manual thematic analysis by the corresponding author (M.S) using software packages Nvivo (20) and Microsoft Office, where themes and subthemes were coded and described. Themes were created through a process of manual familiarity with the data to identify and group responses that were similar. For example, data (i.e., responses) that centered around economic implications would be classified together under a theme labeled “economic implications.” Data within each theme were then further analyzed to identify similarities, and where they existed they were grouped and labeled. For example, within the theme of “economic implications” some responses pertained to perceived expenses in operating cage-free systems as opposed to cage-based systems, and others pertained to a perceived inability to access a market for cage-free eggs that would compensate for any increased operational expenditure. Each of these would be considered sub-themes to “economic implications.” In some instances, responses would be analyzed further again until the data were saturated and labeled into themes to the level in which all similar responses could be grouped, a level of detail as it existed could be reported, and all data were represented. The datapoints (i.e., responses) in each theme and subtheme were then quantified to understand the frequency and, therefore, emphasis according to the producers.

## Results

A total of 224 Asian egg producers were successfully recruited into this study however 22 did not complete the questionnaire. Two hundred and two producers participated through to completion of the questionnaires. Of these, 165 were producers that operate cage systems, and 37 using cage-free systems. This paper focuses primarily on the responses of the cage producers, with an accompanying paper presenting the results of the cage-free producers on the challenges in maintaining cage-free systems, including on-farm operational challenges and the support needed by cage-free egg producers. The numbers of cage producers that participated from each country were opportunistic and were: China (22); Indonesia (103); Japan (10); Malaysia (8); Philippines (10); Thailand (12); with a total of 165 cage producers. Producers' responses are shown below each question, in the order in which they appeared in the questionnaire.

### Perceived reasons to use cage-based systems

“*Most egg farmers in your country and around the world use cages. What are the reasons for using cages compared to cage-free systems?*”

**Figure 1:**
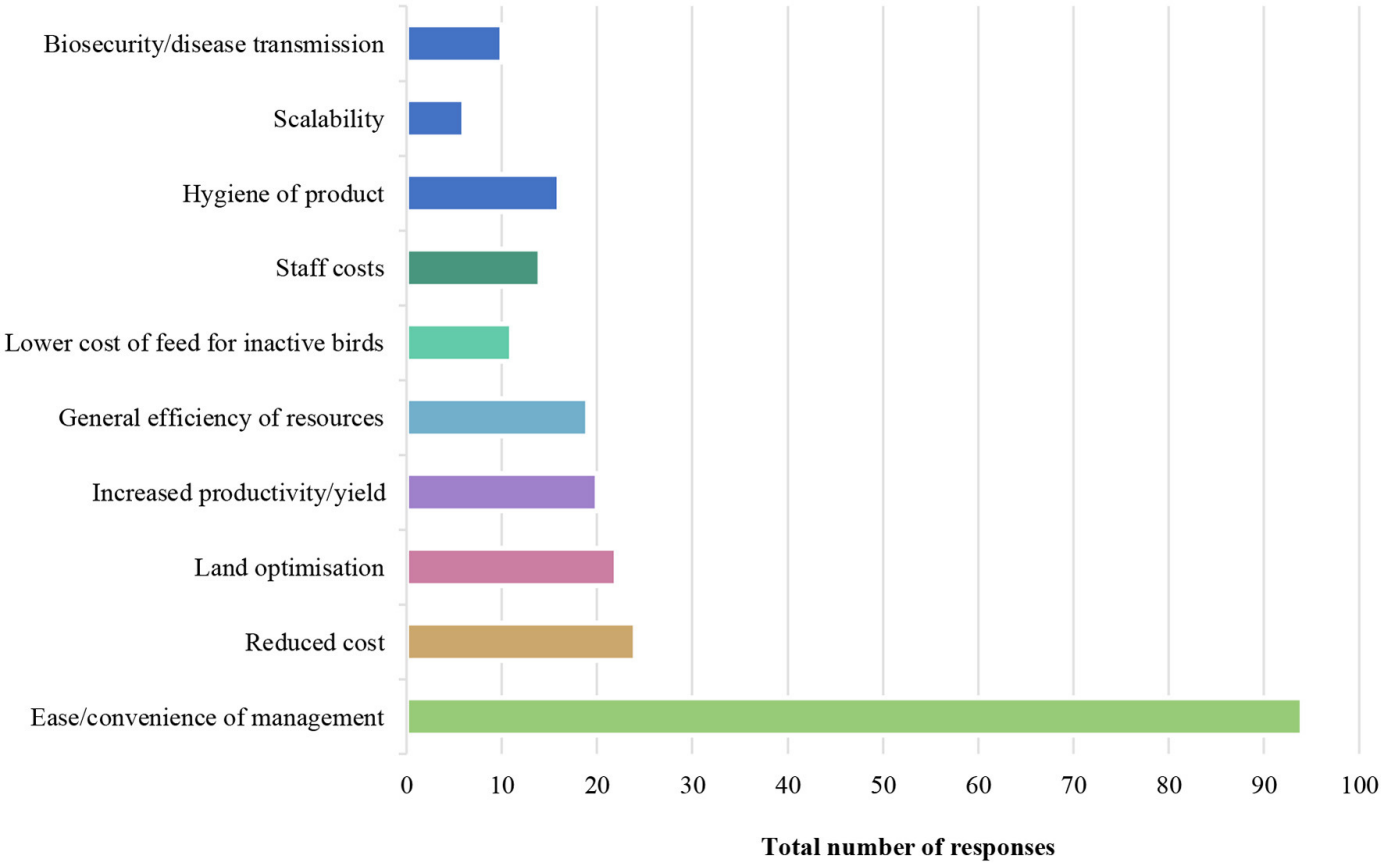
Cage egg producers' top 10 reasons for using cage over cage-free systems of production, displayed as the aggregate of data across all countries.

The convenience of operations and the reduction of costs were the most frequently cited reasons for using cage-based systems, as cited by producers. Summarized responses are listed per country in Table 2, and are displayed as an aggregate across countries in Figure 1.

**Table 2:** Ranking of reasons for using cage systems rather than cage-free systems, by country (cage producers, ***n*** = 165).

	**Themes—number of responses**
China	•Reduce cost (*n* = 9) •Land optimization (*n* = 5) •Ease/convenience of management (*n* = 5) •Scalability (*n* = 5) •Staff costs (*n* = 3)
Indonesia	•Ease/convenience of management (*n* = 45) •General efficiency of resources (*n* = 23) •Land optimization (*n* = 7) •Increased productivity/yield (*n* = 5) •Staff costs (*n* = 2)
Japan	•Hygiene of product (*n* = 6) •Reduced costs (*n* = 4) •Ease/convenience of management (*n* = 2) •Increased productivity/yield (*n* = 2) •Biosecurity/disease transmission (specific emphasis on humidity and moisture mitigation; *n* = 2)
Malaysia	•Increased productivity/yield (*n* = 4) •Reduced cost (*n* = 3) •Ease/convenience of management (*n* = 2) •Land optimization (*n* = 2) •General efficiency of resources (*n* = 2)
Philippines	•Ease/convenience of management (*n* = 7) •Reduced cost (*n* = 4) •Land optimization (*n* = 2)
Thailand	•Reduced costs (*n* = 4) •Ease/convenience of management (*n* = 3) •Staff costs (*n* = 2)
All countries	•Ease/convenience of management (*n* = 94) •Reduced cost (*n* = 24) •Land optimization (*n* = 22) •Increased productivity/yield (*n* = 20) •General efficiency of resources (*n* = 19)

Themes were included when they appeared at least twice in responses within a country's data and were limited to the top five for each country.

### Perceived reasons to adopt cage-free systems

“*Some egg farmers are changing to cage-free systems. What do you think are the reasons to use cage-free compared to cage systems?*”

**Figure 2:**
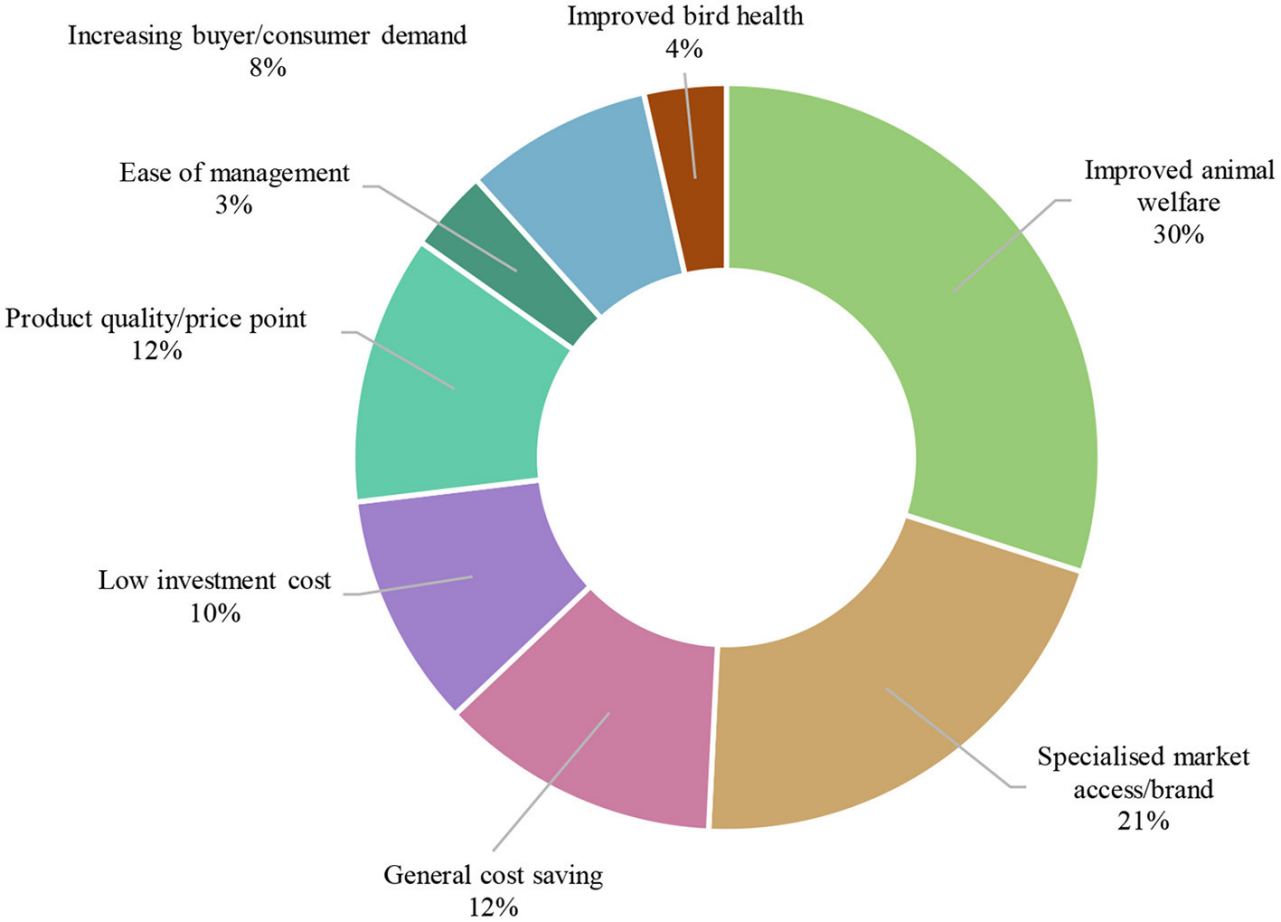
Cage egg producers' perceived reasons to adopt cage-free systems across all countries.

A total of 93.4% cage egg producers identified at least one reason to adopt cage-free systems. Improving bird welfare, gaining access to a wider market, and brand differentiation were the most frequently cited reasons producers identified for using cage-free systems of egg production. All reasons to consider adopting cage-free systems are ranked by frequency of appearance by country in Table 3, and visually displayed as an aggregate across the region in Figure 2.

**Table 3:** Ranking of perceived reasons that cage-egg producers adopt cage-free systems in each country, including frequency of appearance of response (*n*) per country.

**Country**	**Top responses of cage producers by number**
China (*n* = 28)	•Improved animal welfare (*n* = 7) •Increasing buyer/consumer demand (*n* = 5) •Improved product quality (*n* = 5) •Access to higher-end market/higher price point (*n* = 3) •Access to government subsidy (*n* = 2)
Indonesia (*n* = 53)	•Improved animal welfare (*n* = 31) •Low investment cost (*n* = 18) •General cost saving (*n* = 15) •Management improvements (*n* = 8) •Improved bird health (*n* = 4)
Japan (*n* = 14)	•Higher price point (*n* = 6) •Increasing buyer/consumer demand (*n* = 2) •Brand marketing/differentiation (*n* = 2) •Improved animal welfare (*n* = 2)
Malaysia (*n* = 12)	•Improved animal welfare (*n* = 4) •Increasing buyer/consumer demand (*n* = 3) •Access to higher end market/higher price point (*n* = 3) •Brand marketing/differentiation (*n* = 2)
Philippines (*n* = 25)	•Improved animal welfare (*n* = 8) •Access to higher end market/higher price point (*n* = 3) •General cost saving (*n* = 3) •Access to humane “guilt-free” market (*n* = 3) •Access to “health food” market (*n* = 2) •Brand differentiation (*n* = 2)
Thailand (*n* = 26)	•Brand marketing/differentiation (*n* = 7) •Improved animal welfare (*n* = 6) •Access to international markets/keeping up with modern global practices/EU standards (*n* = 3)
All countries (*n* = 158)	•Improved animal welfare (*n* = 59) •Wider market access/increasing demand/brand (*n* = 50) •General cost saving (*n* = 24) •Product quality/price point (*n* = 23) •Low investment cost (*n* = 20)

Themes were included when they appeared at least three times within that country data and are limited to top 5 for each country.

### Perceived feasibility of cage-free systems

“*Do you think cage-free systems are an option in your country? (Yes/No)*”

**Figure 3:**
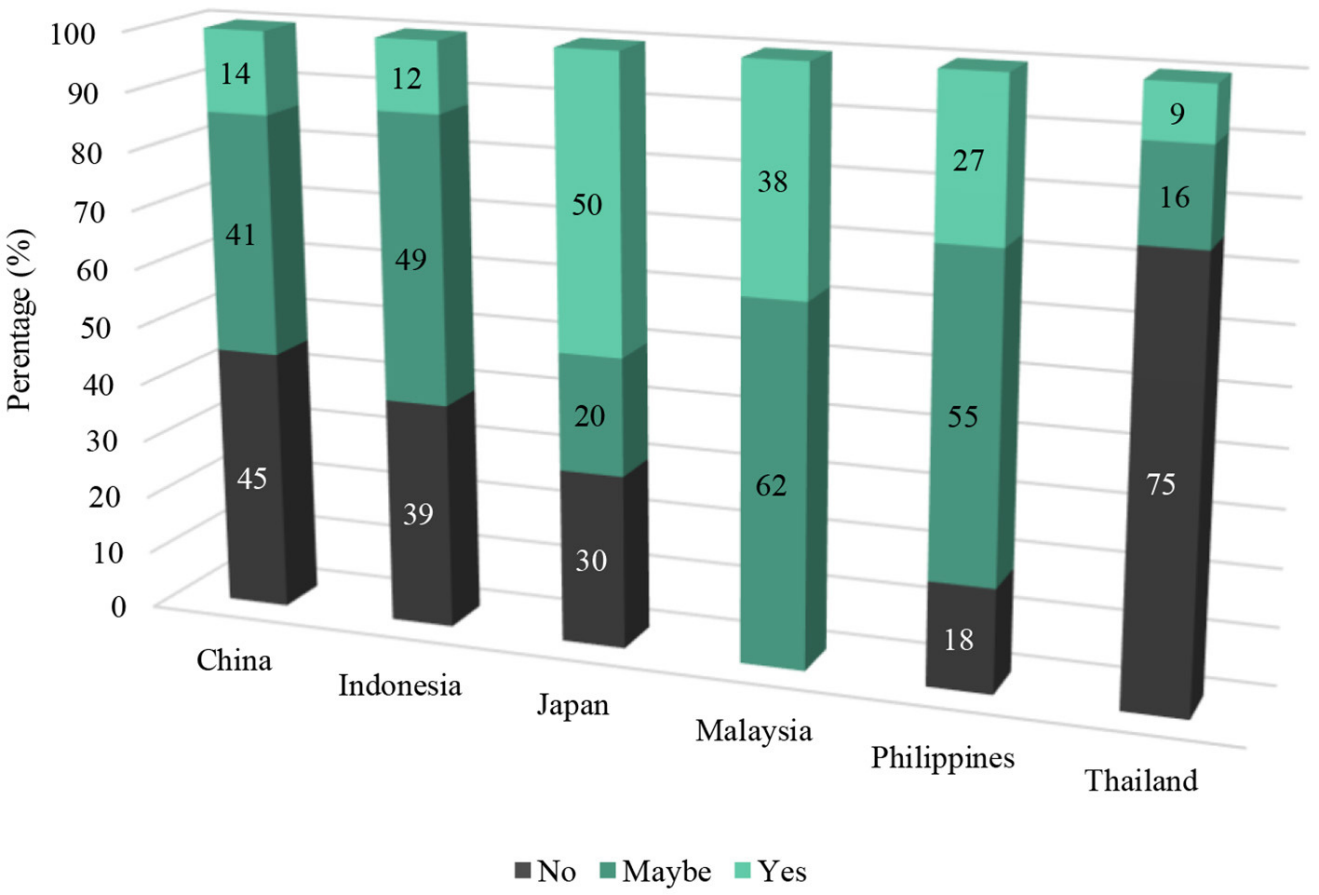
Cage egg producers' perceived feasibility of cage-free systems in their respective countries by percentage (%).

Across all countries, 24.8% of egg producers responded “Yes,” 35.5% responded “No,” and 40.6% responded “Maybe.” The distribution of these responses, by country, are presented in Figure 3.

### Barriers to adopting cage-free systems

“*What do you think are the biggest challenges and problems that prevent cage farmers from using cage-free systems?*”

**Figure 4:**
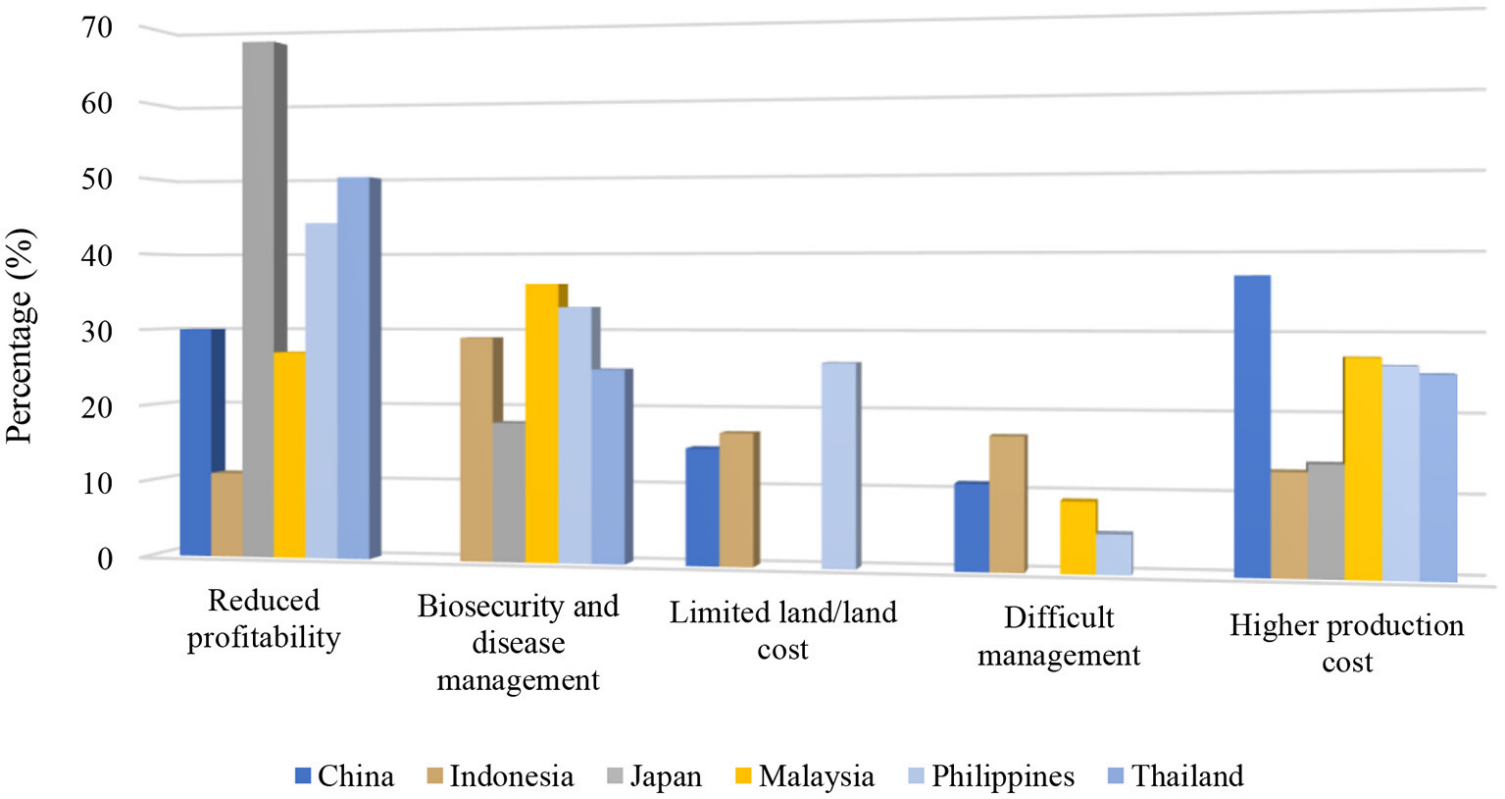
Cage egg producers' most frequently identified barriers to adopting cage-free systems displayed by country.

A total of 217 barriers to moving to cage-free systems were identified by cage producers (*n* = 165). These barriers often represented recurring themes, predominantly centered around land availability, cost, management, and disease mitigation. The themes that emerged through the data, and their quantification, are visually summarized in Figure 4. Themes appearing in > 2% (*n* ≥ 4) of responses were considered notable for inclusion during thematic analysis.

### Solutions to adopting cage-free farms

“*If an egg farmer in your country decided to use a cage-free system what would be some of the solutions to the challenges (outlined above)?*”

**Figure 5:**
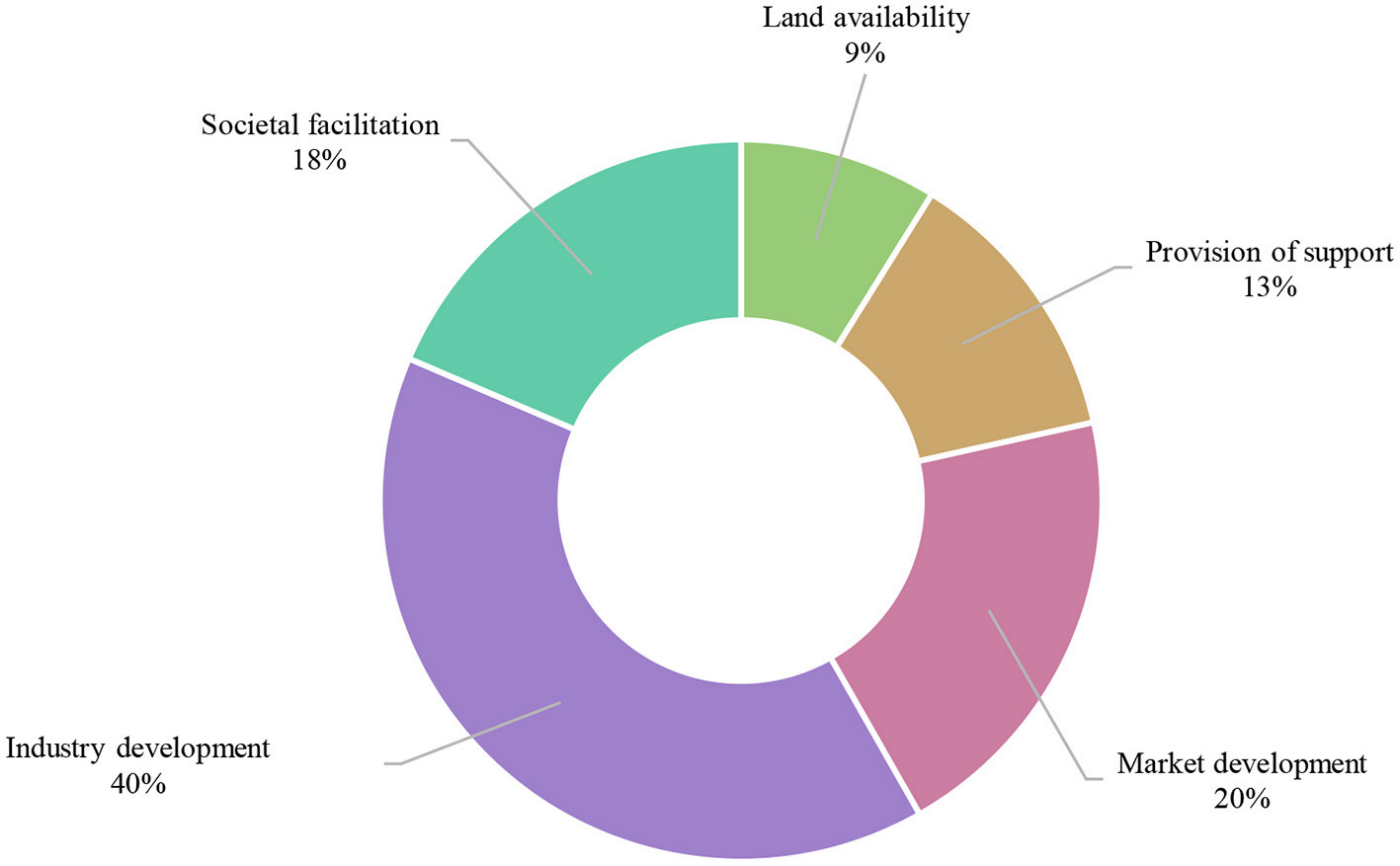
Egg producers' most frequently proposed solutions to the barriers preventing cage farmers from using cage-free systems.

Most commonly, industry development such as the application of technologies in improving on-farm practices and bird health in cage-free systems, along with market development, including demonstration that cage-free eggs can be sold at a higher price, were cited as solutions by egg producers. Quantification of the emerging themes is provided in Table 4, and the top themes are shown in relation to each other in Figure 5.

**Table 4:** Frequency of perceived solutions to overcoming the aforementioned barriers that prevent cage farmers from using cage-free systems.

**Emerging themes**	**Frequency**
**Land availability**	
•Provision or purchase of an appropriate land area	21
•Establish farms further away from the business districts and prevent agricultural land conversation to residential	5
•Establish farms in appropriate environments/climates	5
**Provision of support**	
•Availability of financing/investors	13
•Affordable staff resourcing/Human Resources training	8
•Increase government subsidy/industry incentives	3
•Provision of nests and housing resources	4
•Equipment and maintenance	2
**Market development**	
•Price increase (eggs)	21
•Increase demand/consumption	9
•Demonstrate total increase profit in cage-free farming	7
•Standardize price for cage-free eggs	6
•Strengthen brand strategy/public relations/events	5
**Industry development**	
•Apply technology and innovation to develop improved on-farm practices (bird health and bird security)	20
•Demonstrate effective disease mitigation strategies/biosecurity/food safety	19
•Apply technology and innovation to develop improved general on-farm management practices (including feed distribution, flock sizes, and behavioral management)	16
•Knowledge increase/training for cage-free system planning/demonstrate benefits	13
•Increase productivity and feed conversion ratio	10
•Economic planning/sustainability	4
•Improve added value/quality of products	3
•Enriched cages or barns	3
•Restrict import eggs from overseas	2
•Limit volume of operation	1
•Major buyers take the lead	1
•Improved labeling	1
•Policy support	1
**Societal facilitation**	
•Community education (animal welfare, advantages and pricing)	6
•Introduction of legislation or regulation/all producers on the same system (incl grace period)	4
•More research/Investigate local alternatives that achieve the same results	3
•Continued GDP (gross domestic product)/economic growth	3
•Address more important issues first (i.e., antibiotic use)	1
**Total**	220

Displayed as the number of times the theme appeared in producers' answers.

### Support needed to adopt cage-free systems

“*If an egg farmer decided to use a cage-free system, would they need more support in the establishment or maintenance of the farm than is currently available?*”

Across all countries, 72% responded “yes,” 7% “maybe,” and 22% “no.”

“*What support would they need?*”

When asked to share their thoughts on the nature of support that is needed in considering adoption of cage-free systems of egg production, producers drew attention to the need for training, knowledge and access to experts in effective cage-free operations and bird health, along with financial assistance, including subsidies and capital, and market growth through consumer awareness. For example, one cage producer in Indonesia stated: “The government should eliminate the upper price limit because it can cause disincentive for farmers…farmers are threatened by operational licensing, and standard price is rarely evaluated based on the farm's budget and cost.”

**Figure 6:**
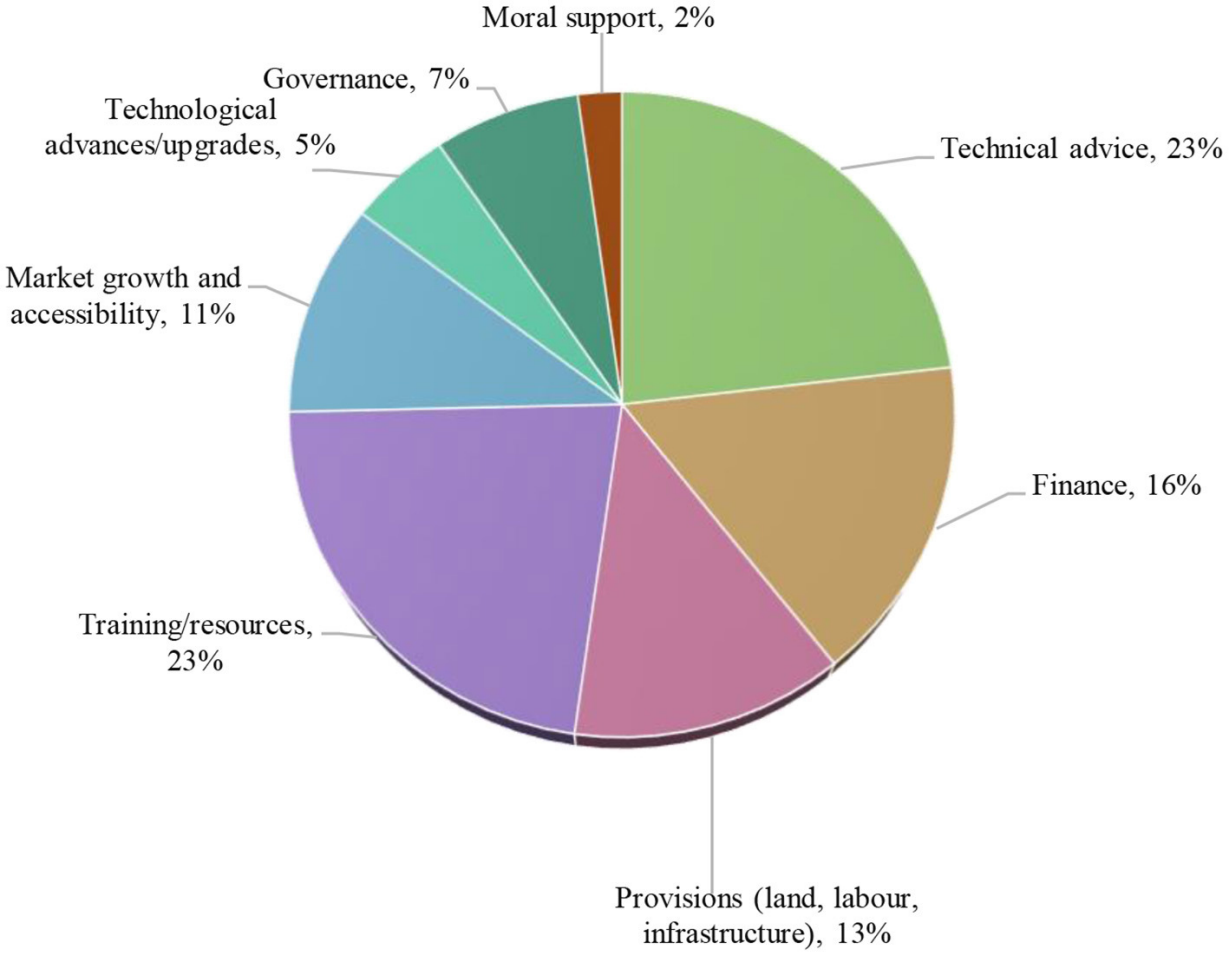
Egg producers' perception of the support needed to transition to cage-free systems, by emerging theme.

The themes of all responses across countries are quantified in Table 5, and an overview is presented visually in Figure 6.

**Table 5:** Frequency of egg producers perception of the support that is needed when looking to adopt cage-free systems in across all countries.

**Emerging theme**	**Emerging sub-themes**	**Frequency**
Technical advice	Efficient operation and management/maintenance	15
	Controlling security/safety/health of birds	13
	Biosecurity/disease	10
	Brand marketing cage-free products	6
	Litter management	3
	Efficient farm layout and design	3
	Feeding management	3
	Shared experiences from other cage-free farmers	1
	Transition process	1
	Weather mitigation	1
Finance	Financial assistance/capital support/subsidies (including loan subsidies)	34
Provisions	Subsidized land (large/suitable)	13
	Staff/labor	12
	Bird provisions (feed, nests, medicine, and litter)	5
	Infrastructure (including roads and electricity)/equipment	4
Training/resources	Share knowledge/technical training for producers and personnel in effective cage-free management (continuous)	37
	Technical support/consultancy (including vets and government, mentors)	13
	Cost-benefit analysis/economic modeling	7
Market growth and accessibility	Grow cage-free market/consumer support through awareness (human health, organic, and animal welfare)	17
	Market accessibility/improve distribution channels (incl. reducing the price of distribution and joint marketing with other cage-free producers)	5
	Consumer acceptance of higher cage-free egg prices	3
Technological advances/upgrades	Advances in disease prevention and control on cage-free farms	5
	Efficiency/productivity upgrades	4
	Advances in egg hygiene/sanitation on cage-free farms	1
	System infrastructure upgrades (i.e., housing)	1
Governance	Law/regulation development	5
	Price regulation/standardization evaluation	5
	Reduced complexity of licensing, establishment of a certification body	3
	Full government support (tangibility, no favoritism)	3
	Policy support (including for trade)	2
Moral support	Understanding/support from the community and local farms (incl. reduced complaints)	4
	Reduce public criticism toward the industry	1

“*Who should offer that support?*”

**Figure 7:**
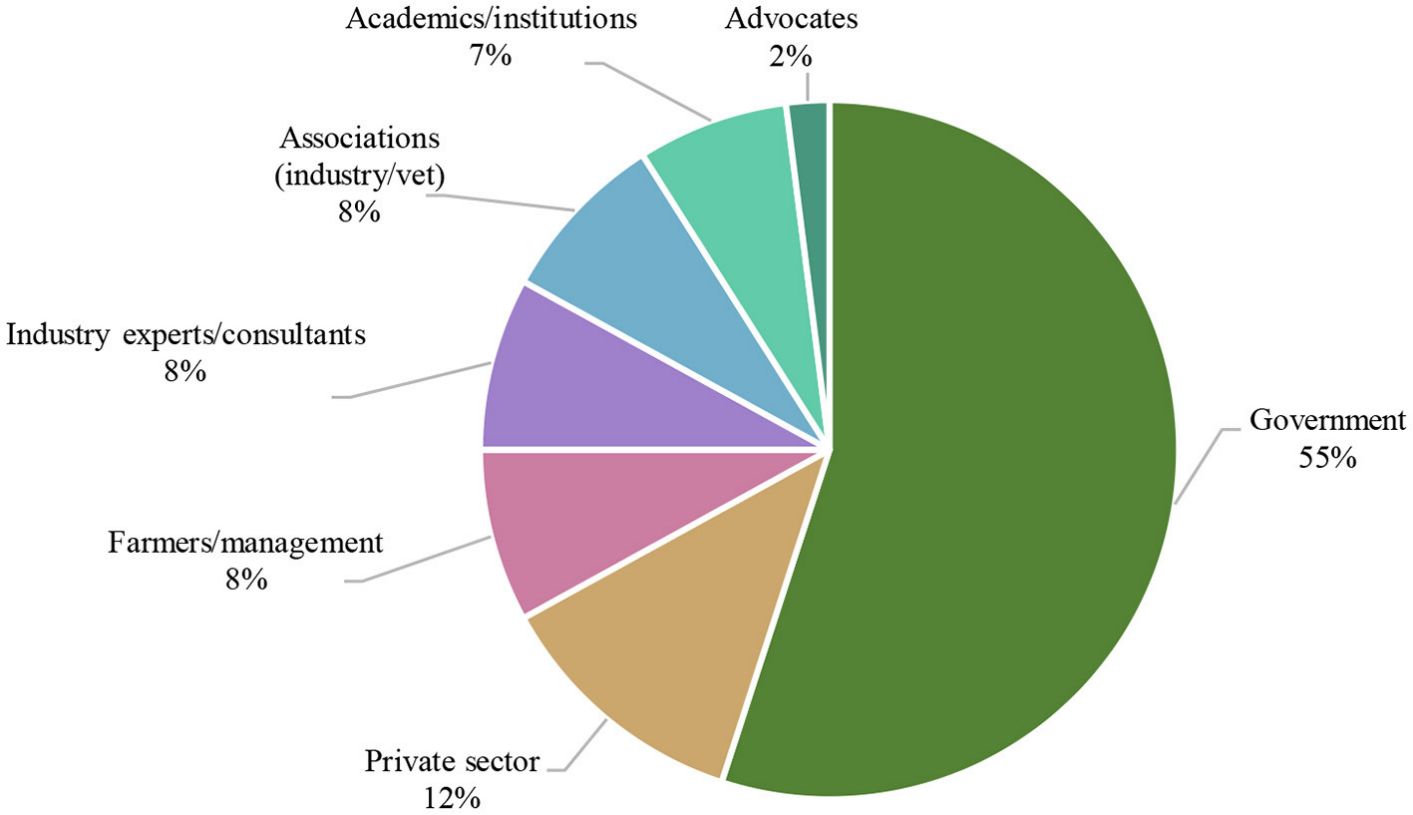
Egg producers' perception of the stakeholders that are most frequently deemed as required to provide support for transitioning to cage-free systems by percentage.

Egg producers most frequently identified their domestic government, and government departments within it (55%), as the stakeholder that should provide support. This was followed by the private sector (12%), then in equal part industry experts/consultants, industry and veterinary associations, and the farming and management network themselves. This data is presented in Table 6, and further illustrated in Figure 7.

**Table 6:** Frequency of egg producers' perceptions around who should be offering the support listed should they transition to cage-free systems.

**Country**	**Responsible party**	**Frequency**
China (*n* = 23)	Government	10
	Professional organizations/Industry	4
	Experts	3
	High end consumers/egg selling companies	2
	Overseas equipment suppliers	1
	Banks	1
	Other countries	1
	Technology service institutes	1
	Unsure	1
Indonesia	Government	48
(*n* = 107)	Academics/institutions	10
	Community/everyone	10
	Related private sector (i.e., systems, bird feed companies, pharmaceutical companies, etc.)	9
	Farmers	8
	Vets/vet associations	5
	Nobody/unsure/unclear	5
	Industry associations	4
	Consultants/specialists	4
	Advocates	3
Japan (*n* = 10)	Government	9
	Nobody/unsure	3
	Private sector	1
	Other cage-free producers	1
	Media	1
Malaysia (*n* = 9)	Department of Veterinary Services (DVS)/Government	6
	Buyers/larger corporation	3
	Universities	1
	Farmers associations	1
	Equipment suppliers	1
	Poultry breeders	1
	Overseas experts	1
Philippines	Bureau of Animal Industry/Government	9
(*n* = 14)	Nobody/unsure	2
	Equipment suppliers	2
	Management	1
	Banks	1
	Related private sector	1
	Other cage-free farmers	1
	Advocates	1
Thailand	Government (Animal Husbandry Department / Department of International Trade/Ministry of Agriculture, Commerce and Public Health)	14
(*n* = 15)	Equipment suppliers	1
	Animal advocates	1
	Media	1
	Banks	1

## Discussion

### Challenges in adopting cage-free systems

The findings of this study present that the main reasons egg producers choose cage systems are centered around efficiency; that they are easier to operate and they reduce costs, while increasing the yield of eggs. Having not been exposed to a natural environment, the eggs are cleaner at collection, reducing cleaning requirements. As is also often the case with intensive housing systems, another incentive for choosing cage-based systems is the ability to utilize land space for maximized output. These perceptions are in line with realities presented in the wider literature, that although relatively comparable in some conditions, cage systems were found to generally be more efficient. One rigorous study in the UK showed that while both cage and cage-free systems met production rate standards published by the National Farmers' Union (21), cage systems produced 5–7% more eggs in the span of a year; a study in Africa showed a difference of battery cage economic efficiency of 0.92 compared to 0.89 in a single level deep litter system (22), and an economic study in India also found efficiencies increased in cage systems (23). These increased efficiencies decrease operating costs. Another recent study in the USA demonstrated that aviary housing system (cage-free) operating costs were 23% higher than conventional cage (battery) systems, while the operating costs for enriched cage systems was 4% higher than conventional (battery) cage systems (24). Increased operating costs feed directly into the top challenge cage producers presented us with in considering a shift to cage-free systems: reduced profitability. On reviewing literature from Europe, North America and Australia, it appears that this is unsurprisingly also the primary reported barrier to transition to cage-free egg production in these regions. An economist's strict analysis of Californian egg prices after the ban of the sale of eggs from battery cage systems found that the prices of eggs increased, which resulted in higher prices and decreased consumer surplus (16). However, when adjusting for available data on the financial value of human altruism, and transversely also adding the transition cost to producers, another study found the opposite in the theoretical case of a nation-wide ban on cage-egg production in the USA; it found that benefits would far outweigh costs (25). Additionally, when considering higher operating costs, some losses could be associated with flock mortality and more generally, lack of experience with efficient cage-free operations. Demonstrating this, one more recent study conducted a meta-analysis of hen mortality across the various systems in 16 different countries over two decades, to find that as experience operating cage-free systems increased, mortality dropped an average of 0.35–0.65% annually, until there were no significant differences between the cage and cage-free production systems (26).

While the above studies are informative in relation to egg production in USA and Europe, they were not conducted in Asia, and findings may not be directly transferable. Agricultural factors often differ across and between regions; including breeds, climate, production systems, availability of farm resources, and other external factors such as the traits of the domestic markets, economic and geopolitical structures, and culture. In this region, the literature has been scarce, with few exceptions. One small but important exception conducted in-depth qualitative interviews with 15 cage egg producers in China. Resonating with findings in Europe and USA this study found that abandoning conventional cages in favor of cage-free systems was considered a financial loss. When this perceived financial loss is coupled with a lack of domestic social pressure to adopt higher welfare systems, interest levels in transitioning to cage-free were unsurprisingly low (3). As echoed in the present findings, it remains that cage systems do present economic incentives to egg producers in Asian countries, as they do around the world. Still, there exists a growing trend to shift away from conventional cages in many global regions, and the current situation and perspectives in the focus countries may change in the coming years. Driving these key developments include domestic and international trends toward higher quality products, and increasing affluence in key states (27). While differences in operational costs and profitability can be demonstrated in present times, the growth of markets willing to offset the welfare of hens, the increasing exposure and experience of producers in relation to cage-free systems, and even the potential for future legislative shifts that ban cage systems, could change this balance considerably. As states find themselves in increasingly comfortable economic positions and stages of development, animal welfare is of increasing concern to consumers (28, 29). In specific regard to the countries investigated within this study, recent research found that of egg consumers in China, Malaysia, Philippines and Thailand, ~72, 73, 77, and 78% in each country respectively stated that it mattered to them that hens laying eggs do not suffer (30). Furthermore, 65, 69, 71, and 68% in China, Malaysia, Philippines and Thailand respectively went on to state that they would prefer to buy eggs from hens not kept in cages (30). This shift is also reflected in the multitude of global commitments from large multinational food companies to source cage-free eggs in their supply chains (14).

The second top challenge identified by producers in the present study, when considering the adoption of cage-free systems, was biosecurity and disease control. To support this, one study found that cage systems did slightly reduce the horizontal transmission of salmonella and campylobacter as compared to cage-free environments on wood shavings (as the shavings were considered to allow the disease to live longer) and cages with manure removal belts slightly reduce the bacteria count on eggs (17, 31). Importantly, however, there was no difference between bacteria on washed cage and cage-free eggs (17). It is important to note that the perspectives presented in this study are the producers' perceptions and are not indicative of consumer perceptions. One example of the potential disparity between perceptions in this study and consumer perceptions was “health benefits” of humans consuming cage eggs. While producers and cage proponents present that the easily monitored and maintained nature of harvesting eggs in cage systems reduces microbiological contact of eggs (32), consumers may instead associate organic, natural and high animal welfare with improved health benefits of the products from cage-free systems (33). Anecdotally, this is also the case with the use of native breeds and traditional farming methods in some areas of Asia, where consumers tend to perceive “naturalness” of these breeds as “healthier.”

In the wider body of literature around challenges to bird health in egg production, destructive hen behaviors—such as feather pecking and cannibalism—are frequently featured, however, these behaviors were interestingly not presented with any significance by egg producers in this study.

Lastly, despite hosting a national land mass at least five times greater than any other country in this study, producers in China (15%) identified the availability of suitable land as a barrier to transitioning to cage-free systems. Most egg production in China (~90%) is cage-based, at a scale seen no where else in the world; ~604.68 billion eggs per year (9). The nation also hosts the greatest population in the world; ~1.4 billion people (34). It is possible that both of these factors impact egg producers' ability to envisage the quantity of chickens currently housed in cages being facilitated in cage-free ranges, alongside the human population.

### Reasons to use cage-free systems

While the majority of egg producers across Asia still use cage systems, the findings of this study demonstrate that producers may be open to cage-free systems through acknowledgment of benefits for their use, and in majority, state that cage-free systems could be feasible in each country. When they were asked if cage-free systems were an option in their country, two-thirds of egg producers responded “yes” or “maybe,” demonstrating a level of openness to cage-free systems. The one exception to this was Thailand, where 75% of producers did not believe cage-free systems to be feasible. The reasons for this were not revealed by this study, however Thailand has a thriving egg industry of over 94.8 million layer hens who are kept predominantly in cages (9).

Importantly, 93.4% of all respondents could identify at least one reason to adopt cage-free systems. The top benefits identified by egg producers in shifting to cage-free systems included improved animal welfare, access to wider markets, brand improvement, improved product quality, and reduced investment costs. While the animal welfare benefits in moving away from conventional cages are well-understood and accepted, additional beneficial aspects such as brand improvement, market widening, and increased sale price have also been demonstrated to grow as consumer awareness grows and cage-free systems become increasingly mandated by buying companies and their governments as a result (35). More broadly, Sinclair et al. (9) found that livestock industry leaders in Asia saw a number of benefits to improving the welfare of animals being farmed in general. These included improved productivity of the animals, improved product quality, reduction in disease, improved food safety and biosecurity, protection of natural resources, improved international trade opportunities, improved brand confidence, and options for increased revenue (9). Contrastingly, “cost savings” was broadly identified as a reason to adopt cage-free while “reduced profitability” was also identified as a challenge to adopting cage-free. In considering benefits more closely, a significant proportion of responses also explicitly identified the cost reduction element of establishing a cage-free farm, as compared to the expenditure required to install cage systems. It is therefore possible that the broader “cost savings” response in considering reasons to adopt cage-free farms, is also referencing this saved infrastructure expense. To consider the potential weighting of the reasons to operate cage vs. cage-free systems, Table 7 compared the top five findings against the results of a previous study with livestock industry stakeholders, which investigated and weighted the importance of general benefits of improving farm animal welfare (9).

**Table 7:** Comparison of the perceived benefits in improving animal welfare in a previous study with livestock leaders in Asia (9) in relation with Asian egg producers in the present study.

Rank^*^	Benefit by “importance” (9)^*^	Comparative benefit “top 10” findings in present study (2022)^**^	
		**Cage**	**Cage-free**
1	Productivity of the animals; Improve quality of meat or animal product <100%>	Increased productivity/yield	Product quality/price point
2	Reduce disease and injury and treatment costs <53%>	Reduce cost	Improved bird health
3	Avoid cruelty and reduce animal suffering <53%>		Improved animal welfare
4	Increased revenue/profit <47%>	Reduce cost Ease/convenience of management Land optimization Scalability General efficiency of resources	Low investment cost Wider market access/increasing demand/brand/differentiation Access to international markets/keeping up with modern global practices/EU standards General cost saving
5	Human health/zoonosis; Protection of natural resources/ecosystem development <35%>	Hygiene of product Biosecurity/disease transmission	

“Rank” indicates a rank in importance across the countries from the findings in the previous study on the generalized benefits of addressing animal welfare in animal agriculture.

* <%> indicates the percentage of focus groups (*n* = 17) in which the listed benefit was presented by livestock leaders.

** <%> indicates the percentage of countries in which the benefit was presented as an important theme.

### Solutions to the challenges

The top barriers for cage producers considering adopting cage-free systems, related to a perceived loss of profitability, increased direct and indirect costs—including disease—and a higher cost of production. This was not surprising, and is in line with literature from other areas of the world (16, 25, 36). The reduced efficiency and profitability that was perceived as a barrier to adopting cage-free farms is in part mitigated by the proposed solutions of development of the industry, market development and increased sales, and an increased price point. Coupled with market growth, improving the efficiencies of cage-free farms through training on best practices, technical advice, and investing to build cage-free efficiencies could also begin to address these challenges. These findings were echoed in a qualitative interview study conducted within China (3, 37), in which cage egg producers also suggested that increasing the domestic demand for higher welfare eggs through marketing, coupled with simultaneous ancillary measures such as exploring appropriate cage-free systems, and introducing regulation and producer training in cage-free system management, would provide solutions to producers desiring a transition to cage-free systems of egg production (3).

Although participants in the present study were not tested on their knowledge around cage and cage-free systems, some remarks and inconsistent responses provided by some cage egg producers could be interpreted as a lack of comprehensive understanding as to what constitutes a commercial cage-free farm (including barn and aviary systems). Awareness around what constitutes cage-free egg farms, and how they can operate effectively on a commercial scale, could be of foundational benefit. The perception of reduced control pertaining to bird health and biosecurity, and the perceived reduced ability to prevent and treat disease, could also be addressed through the demonstration of model farming and biosecurity practices. In addition, applying technology and innovation to address bird health and biosecurity concerns were presented as solutions by producers, which could also be considered reasonable and practicable ways to mitigate concerns with a shift to cage-free systems. Further investigation to identify the specific technologies and technological development that were inferred by producers would be useful.

### Support needed to adopt cage-free systems

Most producers believed that more support is needed to establish cage-free farms. Amongst the top types of support that were deemed needed were technical advice, training and resources. This reflects the findings by another recent study in the region, where livestock stakeholders presented that training and public awareness were amongst the solutions to wider animal welfare concerns for farmed animals (12). It is important to note that whilst cage-free systems offer opportunities to vastly improve animal welfare, they also present some challenges. As noted by one review, “improved animal welfare” needs refinement and consistency in practice; “welfare in cage-free systems is currently highly variable, and needs to be addressed by management practices, genetic selection, further research, and appropriate design and maintenance of the housing environment” (38).

In relation to identifying the key stakeholders from whom support is most needed should an adoption of cage-free systems be undertaken, “government,” and specific government departments were identified in all countries, echoing the findings of earlier studies around motivational forces for animal welfare (11, 39), and international strategy (13). With the ability to provide guidance, resources, and to enact law and binding standards and policy, these findings reinforce the importance of government engagement, investment and, at a minimum, collaboration for any large-scale change to be sustainable.

### Summary of animal welfare implications

The study provides an increased understanding of the egg industry in key Asian countries, as well as important solutions and support needed, nominated by egg producers themselves, when considering adopting cage-free systems of egg production. Since cage-free systems have the potential to enhance animal welfare, information that can be used to improve the competitiveness of these systems and support egg producers is crucial.

Summary of the key results:

- The main reason producers choose to use cages—ease/convenience of management (53% of all responses)
- When cage producers were asked whether cage-free systems are a viable option, 35.5% said “no,” 40.6% said “maybe,” and 24.8% “yes,” and 93% of cage producers identified at least one reason to adopt cage-free systems.
- The top four perceived reasons to go cage-free by cage producers included: animal welfare 30%, market access 21%, cost saving 12%, and product quality 12%.
- The top challenges preventing cage producers from adopting cage-free systems are; reduced profitability, biosecurity/disease, and higher cost of production.
- Top proposed solutions to these challenges are; development of the industry 40%, market development 20%, and societal facilitation 18%.
- Most producers believe more support is needed to establish a cage-free farm; 72% “yes,” 7% “maybe,” and 22% “no”.
- The top types of support that is needed are; technical advice 23%, training/resources 23%, and provisions 13%.
- The top stakeholder that producers nominated that should provide support was the government, in 55% of responses.

### Applications

The findings of this study provide a basis with which to engage with egg producers in the focus countries. In the absence of reformative laws, there exists a need to increase the competitiveness of cage-free systems, and an increase in the perceived benefits in favor of cage-free systems. This is particularly the case regarding efficiency and management processes.

Initiatives aimed at supporting the egg industry through training, knowledge dissemination, and financial assistance may have an increased likelihood of engagement with producers in Asia. Some existing programs applied in other areas of the world could be usefully tailored and introduced to Asia. Examples of this could include Hennovation in Europe; “practice-led innovation supported by science and market-driven actors in the laying hen and other livestock sectors” (40), and the establishment of government partnered industry-based training centers.

Further research quantifying the strengths of the reasons to transition to cage-free systems identified by egg producers in this study could be conducted, as could rigorous efficiency comparisons and economic modeling for best practice operated farms of both systems, in the context of local conditions and breeds.

Informed by the key barriers and solutions presented by egg producers in this study, we suggest potential initiatives to support the transition to cage-free egg production in Asia. Some of the listed potential initiatives may be more strongly supported in certain countries. It is important to note that prior to introducing any of the suggested initiatives, further research should be conducted as to the suitability, feasibility, and approach. In considering the findings of this study, regarding perceived stakeholder support, it is also recommended that initiatives partner with government and the local industry wherever possible. Further research with a wider range of expert stakeholders associated with Asian egg industries (poultry experts such as veterinarians, ethologists, housing, climate and management specialists, nutritionists, breeding companies, along with legal, food safety, retail, and marketing experts) could also be beneficially conducted.

Potential initiatives for stakeholders with the goal of facilitating the competitiveness of cage-free systems of egg production in Asia, as suggested by the perceptions of egg producers in the present study, are presented below.

#### Suggested initiatives

- Conduct robust economic modeling to demonstrate the commercial feasibility of modern cage-free farms.
- Increase the competitiveness of cage-free systems by investigating and refining efficiencies and management practices.
- Build the commercial feasibility of cage-free farms through (1) hosting up-skilling activities for existing cage-free farmers (summits, training programs, peer networks), (2) applying science and technology to improve cage-free systems, (3) apply high-end business and marketing principles to grow the market for cage-free eggs (commercial buyers, consumers, and distribution channels) to increase demand.
- Build awareness in egg industries on the realities of efficiently, well run, large-scale commercial cage-free systems.
- Facilitate collaboration with egg producers and local governments to identify suitable land parcels on which to pilot cage-free growth/land parcel program.
- Partnerships with government and industry associations to offer training programs and industry showcases.
- Establish modern cage-free model farms that exhibit best practice and are demonstrable as economic models conducive to a profitable business.
- Apply technology and innovation to develop improved general on-farm management practices, including bird health, bird security, disease mitigation, feed distribution, flock sizes, and behavioral management.
- Apply science and technology to research and develop an improved feed conversion ratio in cage-free farms in the region.
- Increase knowledge and training for cage-free systems, for example by developing cage-free best practice management training programs and sponsor key stakeholders to attend, with a special focus on effective disease mitigation strategies/biosecurity and food safety.
- Workshop solutions and sponsor research and development into addressing the challenges raised in this study, including financial obstacles, including both internally within a company and externally through investors, banks and government support or subsidies.
- Develop resource hubs on best practice management, biosecurity and disease prevention and treatment on cage-free farms, including up to date information on automation and science.

## Limitations

This study represents an initial explorative study. For this reason, this study is foundational, and should be regarded as useful general information and a platform from which to continue more in-depth studies. While this study does not provide a definitive list of potential benefits and challenges in adopting cage-free systems, it does, however, provide initial insight into the benefits the participating egg producers see as possible and important.

A limitation of this study is the investigatory “wide-net” nature, which was designed to investigate an area that has scarcely been researched previously. There is also a lack of quantification around the strength of each item including, in this case, the reasons to operate the different systems, and each “barrier” and each “solution” identified. Further, the format of the methods meant an inability to further question producers in relation to meanings and details of their answers. Another unavoidable limitation was the need to translate all of the information twice. Furthermore, in some areas there is large variability between farm sizes (e.g., caged-farm size in the Philippines ranged from 15,000 to 900,000 birds). While the aim was to target producers from farms that are sizeable enough to be representative of the industry in each local area, some differences may be found in the operation of farms at varied sizes within this range.

While this study sets a useful foundation, it also provides some advice on conducting further quantitative and qualitative investigations in the region.

## Conclusion

This study aimed to better understand the perceived barriers and potential benefits for the egg industry in considering the adoption of cage-free systems. It also investigated the possible solutions to the barriers. These barriers, benefits, and solutions are discussed, and result-advised applications are suggested. The findings suggest that a multi-faceted approach is needed to overcome the barriers that egg producers face in considering a move to cage-free systems, and in implementing solutions. The substantial list of solutions and support needed presented by producers in this study, represents vast opportunities to develop applications that may carry an increased likelihood of engagement with egg producers, and provide support in the way that support is needed.

## Data availability statement

The raw data supporting the conclusions of this article will be made available by the authors, without undue reservation.

## Ethics statement

The studies involving human participants were reviewed and approved by the University of Queensland Human Ethics Committee. The patients/participants provided their written informed consent to participate in this study.

## Author contributions

MS conceptualized the project, created methodology, coordinated data collection, conducted analysis, and wrote the paper. KH conceptualized the project, created methodology, and contributed to writing the paper. QY, ML, and ZI contributed to methodology, conducted data collection, and edited the paper. AA, SI, RI, and JJ conducted data collection. EL conceptualized the project and edited the paper. JN initiated and conceptualized the study. All authors contributed to the article and approved the submitted version.

## Funding

Tiny Beam Fund contributed funds to this project.

## Conflict of interest

The authors declare that the research was conducted in the absence of any commercial or financial relationships that could be construed as a potential conflict of interest.

## Publisher's note

All claims expressed in this article are solely those of the authors and do not necessarily represent those of their affiliated organizations, or those of the publisher, the editors and the reviewers. Any product that may be evaluated in this article, or claim that may be made by its manufacturer, is not guaranteed or endorsed by the publisher.
